# Innovative Biobased Active Composites of Cellulose Acetate Propionate with Tween 80 and Cinnamic Acid for Blueberry Preservation

**DOI:** 10.3390/polym17152072

**Published:** 2025-07-29

**Authors:** Ewa Olewnik-Kruszkowska, Martina Ferri, Micaela Degli Esposti, Agnieszka Richert, Paola Fabbri

**Affiliations:** 1 Chair of Physical Chemistry and Physicochemistry of Polymers, Faculty of Chemistry, Nicolaus Copernicus University in Toruń, Gagarin 7 Street, 87-100 Toruń, Poland; 2 Department of Civil, Chemical, Environmental and Materials Engineering, Università di Bologna, Via Terracini 28, 40131 Bologna, Italy; martina.ferri13@unibo.it (M.F.); micaela.degliesposti@unibo.it (M.D.E.); p.fabbri@unibo.it (P.F.); 3 National Interuniversity Consortium of Materials Science and Technology (INSTM), Via Giusti 9, 50121 Firenze, Italy; 4 Department of Genetics, Faculty of Biology and Veterinary Science, Nicolaus Copernicus University in Toruń, Gagarina 11, 87-100 Torun, Poland; a.richert@umk.pl

**Keywords:** cellulose derivatives, surfactants, phenolic acids, protection of food, active packaging

## Abstract

In order to develop modern polymer films intended for food packaging, materials based on cellulose acetate propionate (CAP) with the addition of Tween 80 as a plasticizer and cinnamic acid (CA), known for its antibacterial properties, were prepared. It should be emphasized that materials based on CAP combined with Tween 80 have not been previously reported in the literature. Therefore, not only is the incorporation of cinnamic acid into these systems an innovative approach, but also the use of the CAP-Tween80 matrix itself represents a novel strategy in the context of the proposed applications. The conducted studies made it possible to assess the properties of the obtained materials with and without the addition of cinnamic acid. The obtained results showed that the addition of cinnamic acid significantly influenced the crucial properties relevant to food storage. The introduction of CA into the polymer matrix notably enhanced the UV barrier properties achieving complete (100%) blockage of UVB radiation and approximately a 20% reduction of UVA transmittance. Furthermore, the modified films exhibited pronounced antibacterial activity, with over 99% reduction in *Escherichia coli*, *Staphylococcus aureus*, and *Pseudomonas aeruginosa* populations observed for samples containing 2 and 3% CA. This antibacterial effect contributed to the extended freshness of stored blueberries. Moreover, the addition of cinnamic acid did not significantly affect the transparency of the films, which remained high (97–99%), thereby allowing the fruit to remain visible.

## 1. Introduction

The global volume of food waste reached an alarming peak in 2022, with a staggering 1.05 billion metric tons of food wasted across retail, food catering, and household sectors. This figure, as reported by the Food Waste Index Report 2024, represents the highest value in the 14-year history of Food Bank research. A detailed analysis further reveals that common discarded items include bread (52%), fruit (38%), vegetables (36%), and cold cuts (32%). A critical strategy for mitigating this enormous volume of food waste lies in prolonging the shelf life of consumable goods. Packaging plays a pivotal role in achieving this by fulfilling several essential requirements. Effective packaging safeguards food products from detrimental external factors such as water, air, light, microbiological contamination, and dust. Modern packaging design extends beyond mere product protection to actively focus on extending shelf life [1] by introducing active additives such as phenols or terpenes [2], flavonoids [3], organic acids, or essential oils [4]. In response to evolving consumer demands, packaging must offer more than just visual appeal or convenience; paramount among its functions is ensuring the safety and integrity of the products it contains.

A promising solution appears to be biobased active packaging, which incorporates additional components within or on the packaging surface to enhance product protective function. Biodegradable polymers used to develop such packaging include chitosan filled with organic acids, or essential oils [3], chitosan-metal or metal oxide materials [5], cellulose derivatives [6], and polylactide-based materials containing quercetin [7] or other natural extracts [8]. This study focuses on producing active packaging based on cellulose acetate propionate (CAP) polymer, supplemented with Tween 80 as a plasticizer and cinnamic acid as the active ingredient.

Cellulose acetate propionate (CAP), an ester derivative of cellulose, uniquely combines the desirable attributes of cellulose acetate—namely, transparency and hardness—with the flexibility and impact resistance characteristic of cellulose propionate. Like other cellulose esters, CAP can be transparent or semi-transparent and readily colored, making it an excellent candidate for products requiring both durability and aesthetic appeal. Despite these advantageous properties, CAP adoption as a packaging polymer is less prominent than that of cellulose acetate, which has been extensively modified with various active additives such as geranyl acetate [9], tea tree oil [10], or lysozyme [11]. In contrast, CAP has predominantly been explored as a component in polymer blends rather than as a standalone packaging material, thus remaining largely overlooked in this application area [12]. This limited interest may stem from the inherent stiffness and brittleness of films composed solely of pure CAP.

To address these limitations and enhance flexibility, this study incorporated a non-ionic surfactant as a plasticizer. Among the available non-ionic surfactants, Tween 80 emerged as a particularly promising candidate, notably due to its approval for food contact by the Food and Drug Administration (FDA). It is worth emphasizing that the scientific literature on the influence of surfactants on the properties of polymeric systems remains relatively scarce, highlighting a significant area for further research. To date, only the effect of Tween 80 on the mechanical and thermal properties of poly(lactic acid) and cellulose acetate butyrate blends has been studied [13]. Tween 80 was also incorporated into polymeric matrices such as the starch and poly(butylene adipate-*co*-terephthalate) blends [14], corn and wheat starch films [15], sodium alginate/carboxymethyl cellulose films [16], or pullulan-based materials filled with cinnamon essential oil [17].

In order to obtain active packaging, active agents like antimicrobial components and antioxidants, must be incorporated into the packaging system. These active agents function by enhancing the stability of the product. Active packaging systems intentionally either absorb specific substances or release them to food or the environment with which the food remains in contact. Compounds required to achieve such an effect may be incorporated into the packaging material. For this reason, cinnamic acid, compatible with CAP and characterized by antibacterial properties, was used as a promising ingredient of active packaging. Antibacterial properties of cinnamic acid were clearly described in the works of Ordonez [18,19], where cinnamic acid was incorporated into starch and polylactide monolayer and multilayer films. Active films based on sodium alginate and pectin containing cinnamic acid were developed by Tong et al. [20]. Cinnamic acid was also used as an active compound in the chitosan-based films dedicated to the storage of blueberries [21]. Moreover, it should be stressed that the inhibition of *Listeria innocua* growth was observed in the case of poly(vinyl alcohol) films filled with cinnamic acid [22]. For this reason, the development of CAP-based materials with cinnamic acid seems to lead to the formation of promising packaging that is able to extend the shelf life of stored food.

It should be stressed that systems consisting of CAP as the polymer matrix and Tween 80 as the plasticizer—either with or without any active additive—have not been studied. Therefore, the materials obtained in this work, as well as the influence of cinnamic acid on the properties of CAP-based films, represent a novel approach within this research area. The obtained films were tested for their structure, morphology, and key properties from a packaging perspective, including mechanical, antioxidant, and antibacterial properties. In addition, water vapor permeability, UV barrier properties, and the effect of the applied additives on the transparency of the resulting films were determined.

## 2. Materials and Methods

### 2.1. Materials

Cellulose acetate propionate (CAP) (average Mn ~75,000), Tween 80, DPPH, and cinnamic acid were obtained from Sigma–Aldrich (Steinheim, Germany). Chloroform, acetone, ethanol, and calcium chloride were supplied from Avantor Performance Materials Poland S.A. (Gliwice, Poland). 2,2-Diphenyl-1-picrylhydrazyl (DPPH) was purchased from Sigma–Aldrich (Milano, Italy). For the storage experiments, Brightwell variety blueberries, imported from Chile, were utilized.

### 2.2. Fabrication of CAP-Based Materials

**Figure 1:**
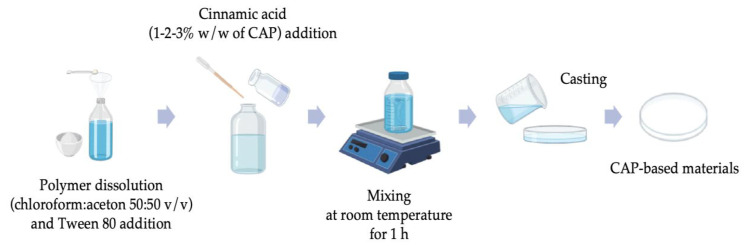
Scheme of the studied film fabrication.

The CAP-based films incorporated with cinnamic acid were obtained by means of the casting method. Polymer (16 g) was dissolved in the mixture (500 mL) of chloroform: acetone (50:50 v/v). In the next step, plasticizer in the form of Tween 80 was introduced into the solution (0.5 g/50 mL). Finally, cinnamic acid (1, 2, and 3% w/w of CAP) dissolved in 5 mL of acetone was added to the CAP-Tween80 solution (50 mL). After 1 h of mixing, the solutions were poured out on Petri dishes and stored at ambient temperature for 48 h. The scheme of the CAP-based film formation has been presented in Figure 1. To identify samples, the following acronyms were used: C–CAP, T-Tween 80, CA-cinnamic acid; 1,2,3 indicate the percentage of cinnamic acid.

### 2.3. Characterization of CAP-Based Films

#### 2.3.1. Fourier Transform Infrared Analysis

Fourier-transform infrared (FTIR) spectroscopy was performed using a PerkinElmer Spectrum Two spectrometer (Llantrisant, UK), equipped with a diamond-attenuated total reflection crystal to analyze the structural properties of CAP-based films, both unmodified and modified with cinnamic acid. All spectra were recorded under the following conditions: 16 scans, a resolution of 4 cm^−1^, and a wavenumber range of 400–4000 cm^−1^.

#### 2.3.2. Morphology and Topography Study

The morphology of the samples was evaluated by scanning electron microscopy (SEM) by observing the top view of the prepared films. Before analysis, samples were metallized with sputtered gold. Investigations were conducted using a Quanta 3D FEG (FEI Company, Hillsboro, OR, USA), and the analysis was conducted by applying an accelerating voltage of 5 or 10 kV. The obtained images were analyzed by Image J open-source software (2.16.0 version). Additionally, atomic force microscopy (AFM) was used to investigate the topography. An atomic force microscope with a scanning SPM probe of the NanoScope MultiMode type (Veeco Metrology, Inc., Santa Barbara, CA, USA) was employed to calculate the roughness parameters as means of three measurements, such as the root mean square (Rq) and arithmetical mean deviation (Ra). Scanned area 10 × 10 µm^2^.

#### 2.3.3. Mechanical Properties

The mechanical properties of the films, including Young’s modulus (E), elongation at break (ε), and tensile strength (σ_m_), were evaluated using an EZ-SX testing machine (Shimadzu, Kyoto, Japan). Measurements were carried out at a crosshead speed of 10 mm/min under a load of 100 N.

#### 2.3.4. Water Vapor Transmission Rate

The influence of cinnamic acid on the water vapor transmission rate (WVTR) was assessed following the procedure indicated in our earlier publication [23]. During the experiment, the weight change of CaCl_2_ was monitored every 24 h over a seven-day period. The tests were conducted at a constant relative humidity of 75% and a temperature of 30 °C. The WVTR was determined using the following Equation (1).

(1) $$WVTR=\frac{rate\;of\;moisture\;absorption\;by\;dessicant}{surface\;area\;of\;the\;specimen}\;\left[\frac{g}{m^{2}\;\times h}\right]$$

#### 2.3.5. Transparency and UV Barrier Properties

The optical properties of CT and CTCA films were analyzed by recording UV-visible spectra within the 200–800 nm range using a Jasco V-650 spectrophotometer (Tokyo, Japan) operating in transmittance mode on specimens measuring 1 × 3 cm^2^. Each spectrum was collected at a scan speed of 400 nm·min^−1^ and a resolution of 0.5 nm. The transparency of each film was determined from the transmittance values at 600 nm (T_600nm_). The UV-blocking activity was calculated as follows [24]:

(2) $${UVX}_{blocking}=100-T_{UV-X}$$

where T_UV-X_ are the average transmittance values in the corresponding spectral regions, specifically UV-A from 400 nm to 315 nm and UV-B from 315 nm to 280 nm. The results were averaged, and standard deviations were calculated from two measurements for each sample.

#### 2.3.6. Assessment of Antioxidative Properties

The radical scavenging activity of materials based on CAP with and without the addition of cinnamic acid, reflecting their antioxidative properties, was assessed using the DPPH free radical scavenging method. Measurements were conducted with a UV-visible spectrophotometer (V-650, Jasco, Tokyo, Japan) in absorbance mode. Absorption spectra of a 0.2 mM DPPH solution in ethanol (3 mL) were recorded over time, both with and without film samples (0.5 × 1.0 cm strips). Data collection occurred at the following intervals: 15, 30, 45, 60, 90, 120, 180, 240, 300, 360, 420, and 1440 min. Spectra were collected across 200–800 nm. The radical scavenging activity (RSA%) was calculated using the following Equation (2):

(3) $$RSA\%=\left(1-\frac{A_{t_{x}}}{A_{t_{0}}}\right)\cdot 100$$

where A_tx_ is the maximum absorbance at particular time intervals after the introduction of the films, and A_to_ is the maximum absorbance at t_o_. Results represent mean values from three independent sample measurements.

#### 2.3.7. Blueberries Storage and Firmness

**Figure 2:**
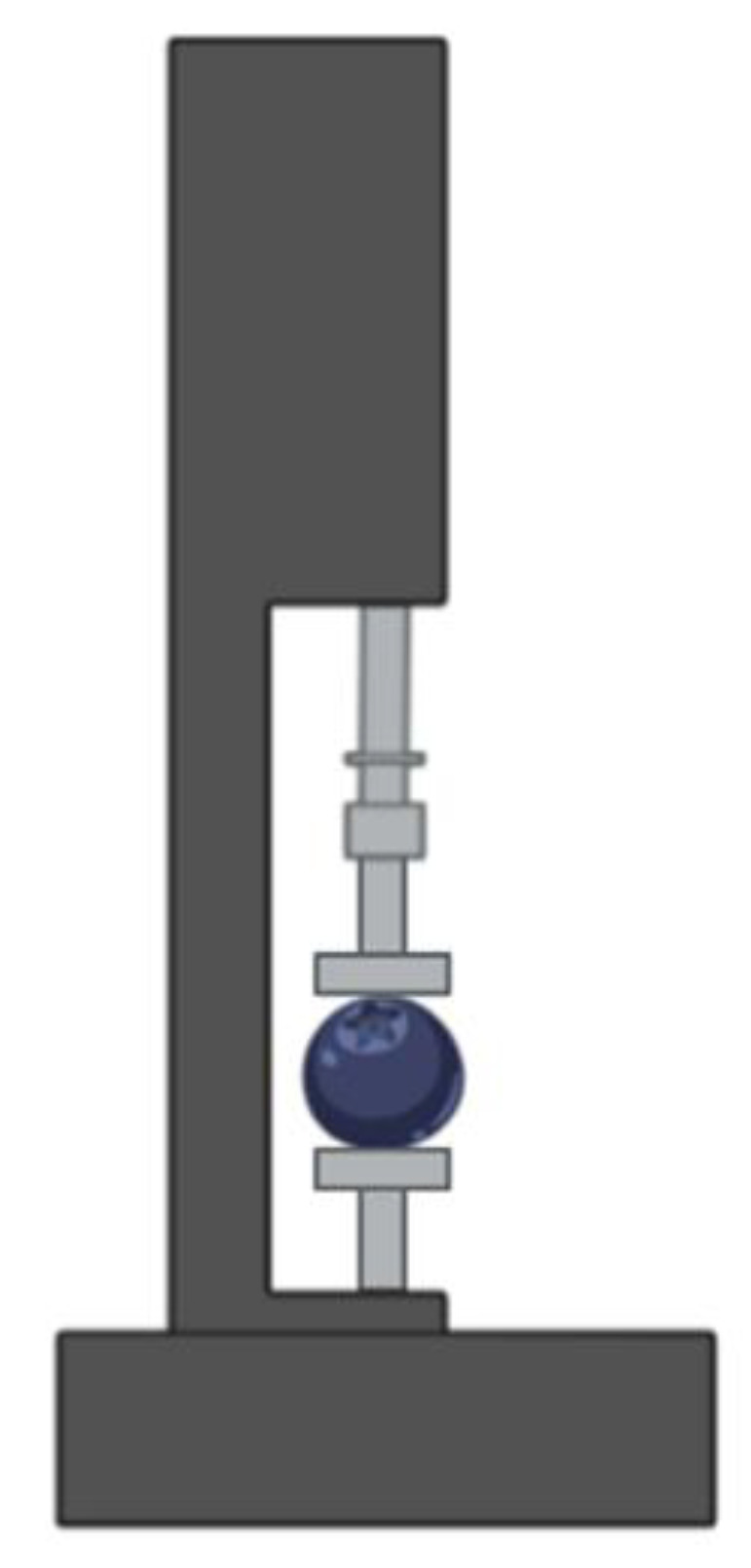
Scheme of the blueberries compression.

Fresh blueberries, free from visible physical damage, were selected and packed in the tested polymer films containing cinnamic acid, as well as films without any additives. Two packages were prepared for each type of film, with each package containing four blueberries. The formed packages were stored for seven days under the same conditions as those used for the WVTR measurements. The weight of the packages was measured every 24 h to monitor the mass loss of the packed fruit. The mechanical properties of the blueberries were evaluated both before and after the seven-day storage period. Mechanical tests were conducted using an EZ-SX texture analyzer (Shimadzu, Kyoto, Japan). A cylindrical probe (20 mm in diameter) (Figure 2) was used for the analysis. During the test, a 20% deformation of the blueberries was applied, and the force required to achieve this deformation, as well as the Young’s modulus, were determined.

#### 2.3.8. Determination of Bactericidal Properties

*Antimicrobial Activity Using the ISO 20645 Method*

To qualitatively evaluate the antimicrobial activity of the tested materials, the agar diffusion plate method was employed in accordance with ISO 20645:2004 standard (“Textile fabrics—Determination of antibacterial activity—Agar diffusion plate test”). This method is based on observing the inhibition of bacterial growth around a sample placed directly on the surface of an agar medium inoculated with a microbial suspension.

Typically, model bacterial strains such as *Staphylococcus aureus* (ATCC 6538), *Escherichia coli* (ATCC 11229), and *Pseudomonas aeruginosa* (ATCC 8739) are used. The test material (e.g., polymeric film, coated textile, or functional composite), usually in the form of a 25 mm diameter disc, is placed on the inoculated agar surface. The plates are then incubated for 18–24 h at 37 ± 1 °C. After incubation, the plates are evaluated for the presence and size of any inhibition zone surrounding the sample and for bacterial growth directly beneath it. The results are classified qualitatively based on the presence and extent of growth inhibition. A clear zone around the sample indicates diffusive antimicrobial activity (e.g., due to the release of a biocidal agent), whereas inhibition limited to the area directly under the sample suggests contact-based activity. ISO 20645 is widely used for preliminary screening of antibacterial properties, particularly in studies of textiles, polymer coatings, and functional surface treatments (ISO 20645).

*Antibacterial Activity According to ISO 22196 Method*

To assess the antibacterial activity of the investigated polymeric materials, the ISO 22196:2011 standard (“Measurement of antibacterial activity on plastics and other non-porous surfaces”) was applied. This standardized method provides a quantitative assessment of the ability of non-porous surfaces to inhibit the growth of bacteria and is commonly used in studies involving biocidal polymeric materials.

The procedure involves applying a suspension of model microorganisms, typically *Staphylococcus aureus* (ATCC 6538P) and *Escherichia coli* (ATCC 8739), *Pseudomonas aeruginosa* (ATCC 8739), onto the surface of the test sample, which is then covered with a polyethylene film to ensure uniform contact and to prevent drying. Samples are incubated for 24 h under controlled conditions of temperature (35 ± 1 °C) and relative humidity (≥ 90% RH). After incubation, bacteria are recovered from the surface, and the number of viable cells is determined by serial dilution and plating on appropriate culture media. The antibacterial activity is expressed as the difference in the logarithmic number of colony-forming units (CFU) between the untreated control and the sample containing the active component. A reduction (R) of ≥ 2 log units (i.e., ≥ 99%) is generally considered indicative of significant antibacterial efficacy. The application of ISO 22196 in the evaluation of polymeric materials enables a reliable and comparable assessment of the effectiveness of various biocidal additives and their impact on the functional properties of the final products (ISO 22196). Table 1 summarizes the abbreviated methodology of ISO 20645 and ISO 22196 standards.

#### 2.3.9. Statistical Analysis

To assess whether differences between films containing various concentrations of cinnamic acid were statistically significant (*p* ≤ 0.05), a Student’s *t*-test was performed. The null hypothesis stated that there was no difference between the groups. If the calculated t-value exceeded the critical value from the statistical table, the null hypothesis was rejected, indicating a significant difference between the samples (Different letters indicate significant (*p* ≤ 0.05) differences between samples (CT-a, CTCA1-b, CTCA2-c, CTCA3-d)

## 3. Results and Discussion

### 3.1. FTIR Analysis of Studied Materials

**Figure 3:**
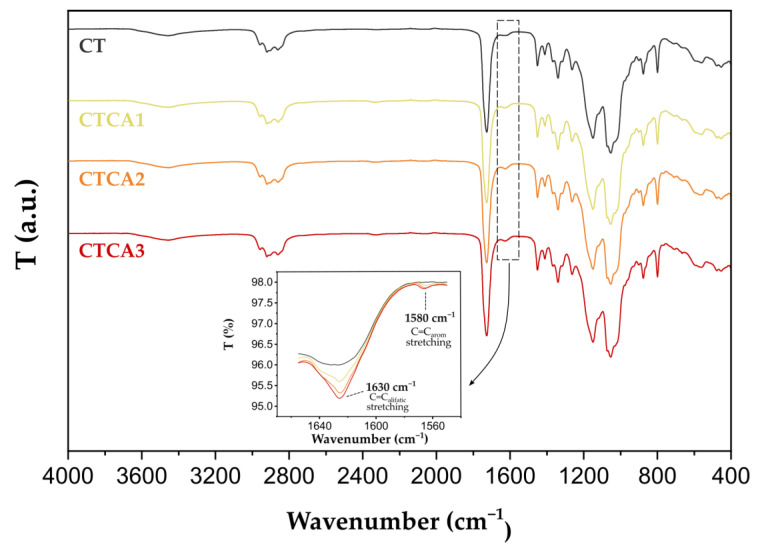
FITR spectra of CAP-based materials. Inset: FTIR spectra of CAP-based materials in the range 1670–1540 cm^−1^.

In order to determine the influence of the presence of cinnamic acid on the structure of the studied materials, an analysis using the FTIR technique was carried out. The obtained spectra are presented in Figure 3. They show bands characteristic of CAP, Tween 80, and cinnamic acid. A broad, intense band with a maximum at around 3480 cm^−1^ indicates the presence of hydroxyl groups (-OH). This band is characteristic of Tween 80 [25], as well as cinnamic acid [20]. It should be noted that the intensity of this band increases with the increasing content of cinnamic acid in the material. The intense band between 2984 and 2884 cm^−1^ belongs to the symmetric and asymmetric vibrations of the -CH_2_ and -CH_3_ groups present in the components of the studied films. Other intense bands visible in the spectra in the range of 1715–1760 cm^−1^, 1000–1100 cm^−1^, and 1230–1240 cm^−1^ correspond, respectively, to the C=O, C-O, and asymmetric stretching vibrations of the C-C-O group [26,27,28,29]. Vibrations in these regions are characteristic of esters and are also present in the structure of all components of the analyzed systems. In addition, the spectra show bands originating from methyl groups present in the ester fragments of CAP, namely acetate esters at around 1370 cm^−1^ and propionate esters at 1270 cm^−1^ [30].

As a result of introducing cinnamic acid into the CT system, two new bands have been recorded at 1630 and 1580 cm^−1^ The band at 1630 cm^−1^ can be attributed to the stretching vibrations of aliphatic C=C, while the band at 1580 cm^−1^ hints at stretching vibrations of the C=C system in the aromatic ring of cinnamic acid [20,29]. The rising intensity of the bands in this spectral range indicates an increasing amount of cinnamic acid. It should, however, be emphasized that no new bands appeared that could indicate the formation of new bonds between the components of the studied systems. This, in turn, leads to the conclusion that they form a physical mixture.

### 3.2. SEM and AFM Results and Discussion

**Figure 4:**
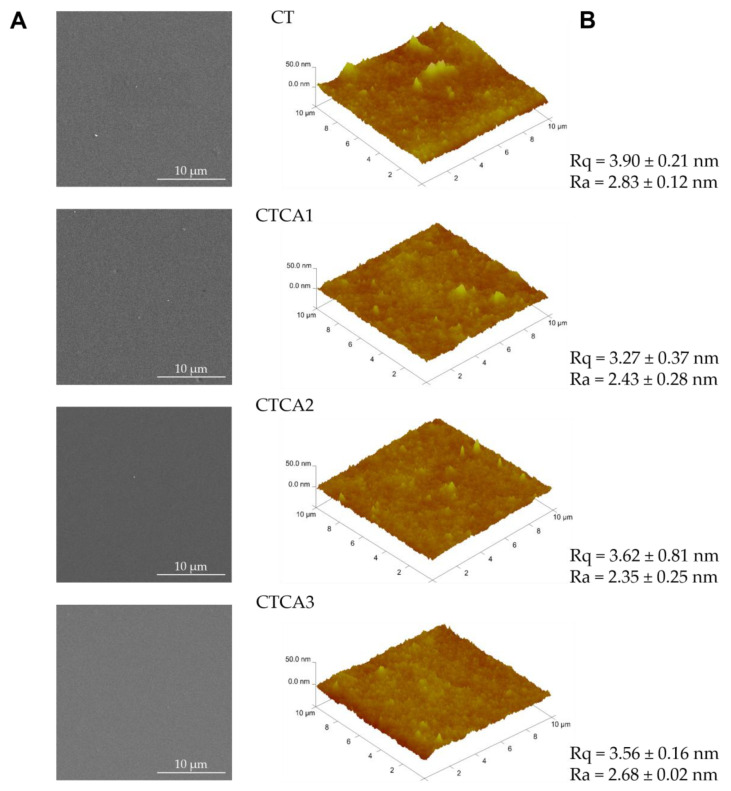
(**A**) SEM (secondary electrons) and (**B**) AFM images of CT, CTCA1, CTCA2, and CTCA3.

It is widely recognized that integrating SEM and AFM enables the analysis of the morphology and topography of polymers containing active components, which is essential for the design of advanced materials. The top-view morphologies of the prepared films were studied through SEM. Figure 4A shows a smooth and uniform surface without irregularities. This evidence suggests a good distribution of the cinnamic acid particles within the matrix without the formation of cracks during the solvent evaporation. Topographic AFM analysis (results reported in Figure 4B) confirms these observations. Rq and Ra values remain almost constant after the addition of the active filler, indicating that the solid particles are perfectly embedded within the polymeric matrix. The same effect of cinnamic acid on the morphology and the topography was observed in the case of materials based on chitosan [21].

### 3.3. Changes in Mechanical Properties

**Figure 5:**
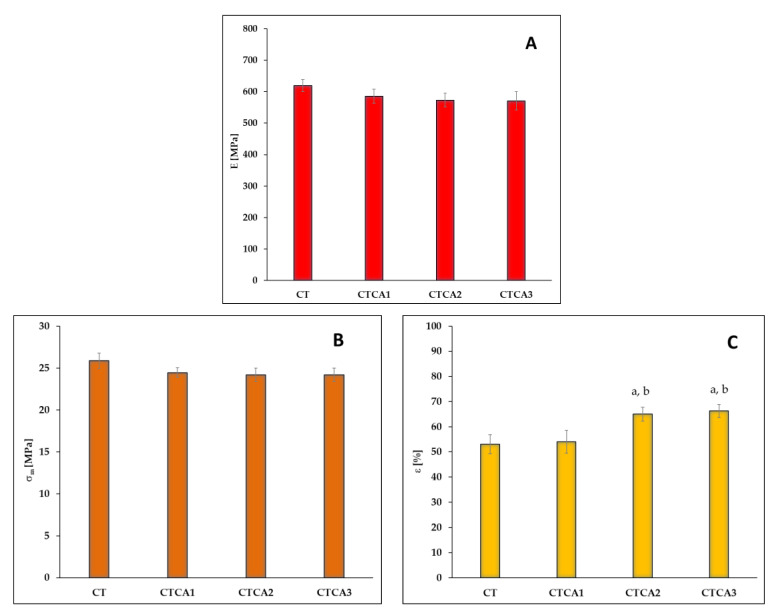
(**A**) Young’s modulus, (**B**) tensile strength at break, and (**C**) elongation at break of the studied materials. (Different letters indicate significant (*p* ≤ 0.05) differences between samples (CT-a, CTCA1-b, CTCA2-c, CTCA3-d)).

Additives introduced into a polymer can interact with it in either a chemical or physical manner. Chemical interactions lead to reactions between the additive and the polymer, resulting in processes such as cross-linking or structural stabilization, thereby protecting the polymer, for example, against degradation. Additives that interact physically are most often dispersed within the polymer matrix and influence the properties of the system without the need for a chemical reaction to occur. Examples of such additives include plasticizers or active packaging components, whose function is to extend the shelf life of food products. In the present study, the effect of cinnamic acid addition on properties crucial for food packaging, including mechanical properties, was analyzed. Figure 5 presents the three main mechanical parameters most commonly discussed for the studied polymer materials: Young’s modulus (E) (Figure 5A), tensile strength at break (σ_m_) (Figure 5B), and elongation at break (ε) (Figure 5C). Based on the obtained data, it was found that regardless of the amount of cinnamic acid introduced into the CT matrix, a slight decrease in both Young’s modulus and tensile strength at break is observed; however, this decrease is not statistically significant. For these two parameters, it can be assumed that the component determining both the stiffness of the material and the stress required to break the samples is the plasticizer, Tween 80. It was introduced into the polymer matrix in a significant amount, which is why the addition of cinnamic acid to the CT system does not notably affect the values of the discussed parameters. An interesting correlation, however, was observed for elongation at break. The addition of cinnamic acid to the CT system does not significantly affect elongation at break, but the introduction of a 2–3% additive causes a statistically relevant increase in elongation at break. It can therefore be assumed that introducing larger amounts of cinnamic acid, which has a much lower molar mass than Tween 80, into a material composed of cellulose acetate propionate (CAP) and Tween 80 can significantly influence the elongation at break. Cinnamic acid, due to its small molecular size and relatively simple structure, is able to more easily penetrate and position itself between the CAP polymer chains. This results in an increased separation of the polymer chains, as the cinnamic acid acts as an efficient plasticizer, enhancing the free volume and mobility within the polymer matrix. As a result, the reduction of intermolecular forces between the CAP chains makes the material more flexible. Compared to Tween 80, which is a much larger molecule, cinnamic acid can intercalate more uniformly between the polymer chains, leading to a more pronounced plasticizing effect per unit mass. Therefore, by increasing the proportion of cinnamic acid in the CAP-Tween80 blend, it is possible to achieve a greater separation of the polymer chains than with Tween 80 alone, resulting in a material that is more flexible and exhibits a higher elongation at break. However, it is important to note that this effect has its limits, as excessive plasticization may eventually lead to phase separation or a loss of mechanical integrity.

An increase in elongation after the addition of cinnamic acid was also observed in the study by Tong et al. [20], where materials based on sodium alginate and pectin were investigated. Similarly, in the study by Zasada et al. [21], as in the case of the materials studied here, it was found that the introduction of cinnamic acid into chitosan did not affect the tensile strength at break but caused a significant increase in the elongation at break of the tested materials. Moreover, it should be noted that cinnamic acid not only can increase flexibility, but its derivatives are also known as plasticizers for polymer materials such as polylactide [31] or poly(vinyl chloride) [32].

### 3.4. Barrier to Moisture

**Figure 6:**
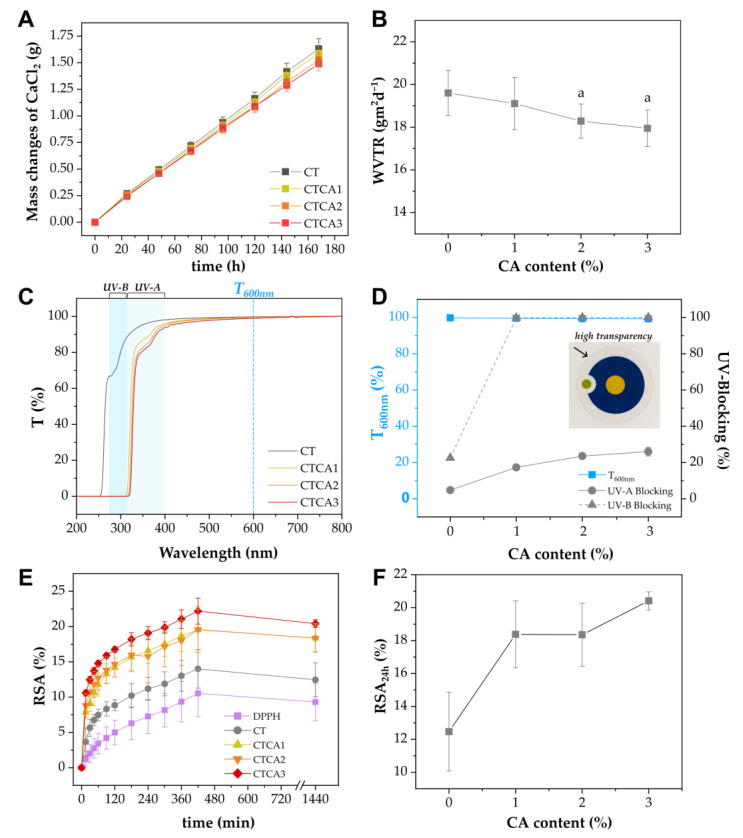
(**A**) CaCl_2_ weight change as a function of time; (**B**) WVTR of the CT and CTCA formulations; Symbol “a” represents *p*-value < 0.05 with respect to the control sample CT; (**C**) Transmittance of CT and CTCA films in the UV–vis range. T_600nm_, UV-B, and UV-A ranges are highlighted in blue; (**D**) Transmittance at 600 nm and UV-blocking properties (calculated according to Equation 2) of CT and CTCA films as a function of CA content. The inset shows the final highly transparent appearance of CTCA3 on a digital logo. (**E**) Radical scavenging activity as a function of time of the CT and CTCA films. DPPH solution without the addition of the films is also reported as a control. (**F**) Final RSA% (at 24 h) obtained by DPPH method as a function of CA content.

One of the key functions of food packaging materials is to serve as a barrier to moisture, preventing either the absorption or loss of water, which can significantly impact the quality and shelf life of the food [33]. To assess the water vapor permeability of CT and CTCA films, changes in the mass of calcium chloride (CaCl_2_) over time were monitored (Figure 6A), and corresponding water vapor transmission rate (WVTR) values were calculated using Equation 1 (Figure 6B). Notably, Figure 6A shows a decreasing slope in CaCl_2_ mass gain with increasing cinnamic acid content. This trend corresponds to the WVTR values in Figure 6B, which reveals an 8.5% reduction from CT to CTCA3 (*p* < 0.05).

Although modest, this reduction suggests that the incorporation of cinnamic acid into the CAP matrix enhances the water vapor barrier properties of the material. This improvement is likely due to hydrogen bonding interactions between -COOH groups of cinnamic acid and the polymer matrix [34], as well as the presence of the aromatic phenyl group in the additive, which can increase the tortuosity of the water vapor diffusion path, as previously reported in other polymeric systems containing additives bearing aromatic structures such as triphenyl acetic glyceroate [35], or tea polyphenols [36].

### 3.5. UV-Blocking Study

In addition to their protective functions, food packaging materials are often designed to provide high optical transparency to meet consumer expectations by allowing clear visibility of the product. At the same time, strong UV-blocking capability is essential to protect the food from ultraviolet radiation, thereby extending its shelf life. Figure 6C displays the transmittance spectra of CT and CTCA films. All samples demonstrated high transmittance across the visible light spectrum, indicating that transparency, calculated by transmittance at 600 nm (T_600nm_), remains unaffected even at the highest cinnamic acid (CA) content. In contrast, within the UV region, highlighted in blue (UV-B) and light blue (UV-A), a progressive decline in transmittance is observed, beginning at 400 nm and becoming more pronounced below 350 nm. This suggests enhanced UV-blocking capability with increasing CA concentration. A summary of T_600nm_, along with the UV-A and UV-B blocking efficiencies, is presented in Figure 6D. Importantly, the incorporation of 3% CA significantly improves the UV-blocking performance by approximately 550% in the UV-A region and 355% in the UV-B region compared to neat CT, without diminishing the material’s transparency (as visually confirmed in the inset of Figure 6D). These findings indicate that CA can be effectively used to enhance the UV protective properties of the films while preserving visual clarity, making CTCA3 a promising candidate for food packaging applications that require prevention of food deterioration by UV-light while keeping the possibility to clearly see the foodstuff through the packaging.

### 3.6. Antioxidative Activity

Beyond simply shielding food from external damage, modern active packaging is designed to interact beneficially with its contents, helping to lengthen food shelf life. Among the advanced functions investigated, antioxidant capacity is a particularly important one. Indeed, by quenching free radicals, the packaging slows down oxidative reactions that could degrade food’s flavor, color, aroma, and nutritional value [37].

In this study, the radical-scavenging activity (RSA%, calculated by Equation 3) over time of CT and films filled with cinnamic acid was investigated through the DPPH assay. The antioxidant activity of cinnamic acid is associated with its ability to neutralize free radicals and protect cells against oxidative stress. Cinnamic acid, similarly to its derivatives, donates a proton to a free radical, accepting its unpaired electron and forming a stable, low-reactivity phenoxyl radical. As a result, it inhibits the formation of reactive oxygen species induced by various factors (including UV radiation), thereby reducing oxidative stress and the resulting damage to lipids, proteins, and DNA [38].

Results of the antioxidant activity of CT and CTCA films are reported in Figure 6E. As expected, the graph shows that the RSA of all samples increases with time. Compared to radical DPPH, measured as control due to its typical self-decay, the slight increase shown by the RSA of neat CT may be attributed to the physical adsorption of DPPH radicals rather than chemical scavenging, a phenomenon previously reported in other polymeric matrices tested under similar conditions [39].

On the other hand, when cinnamic acid is introduced into the films, a slight increase in RSA can be observed, with an increasing trend as a function of the cinnamic acid content. In particular, Figure 6F shows that RSA after 24 h increases from 12% of the RSA of neat CT to 21% of the RSA for the CTCA3 sample. This modest yet consistent improvement can be attributed to cinnamic acid’s conjugated structure, which offers some ability to stabilize free radicals, though typically less pronounced than some of its derivatives bearing hydroxyl substituents [40]. However, even a slight improvement in antioxidant performance can contribute to delaying oxidation-related spoilage processes, particularly in systems where transparency and other material properties must be preserved.

### 3.7. Blueberries Storage

Packaging with antibacterial and antioxidant properties represents a modern solution that actively protects food from spoilage, thereby extending its shelf life and reducing losses at every stage of the supply chain. Packaging with antibacterial properties works by eliminating or inhibiting the growth of microorganisms such as bacteria, molds, or yeast, which are the main causes of spoilage in fresh fruit. In the case of packaging with antioxidant properties, the oxidation process and the associated deterioration in fruit quality are inhibited [41,42,43,44]. In the present study, the fruit used to test the effect of cinnamic acid in packaging was blueberries. Blueberries have a positive impact on human health; they improve memory, reduce the risk of hypertension, support sight, and aid digestive processes [45]. Prolonged storage, however, leads to the development of fungi and the eventual decomposition of the products [46].

**Figure 7:**
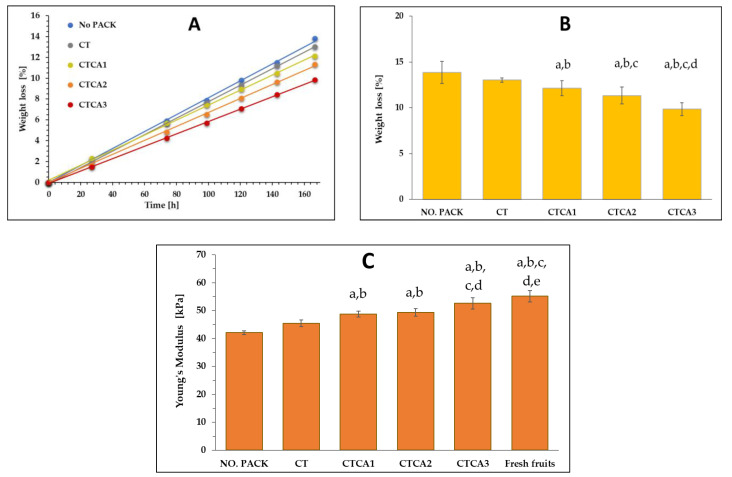
(**A**) Weight change of blueberries during storage, (**B**) Differences in mass loss after 7 days of storage, (**C**) Young’s modulus of fresh and stored fruits. (Different letters indicate significant (*p* ≤ 0.05) differences between samples (NO PACK-a, CT-b, CTCA1-c, CTCA2-d, CTCA3-e)).

The impact of packaging on the packed food was analyzed based on changes in the mass and firmness of the blueberries. Figure 7A shows the percentage change in fruit mass loss during storage, while Figure 7B presents the comparison of mass loss of fruits after 7 days of storage. Prolonged storage time results in a noticeable decrease in the mass of blueberries, ranging from 9 to 14% of the initial mass. The highest mass loss was observed in fruit stored without packaging, indicating that packaging serves as a significant barrier to water evaporation from the blueberries. The introduction of cinnamic acid into the CAP-based materials significantly reduced mass loss in the stored fruit. The smallest mass loss was observed for blueberries packed in a film containing the highest amount of cinnamic acid. This suggests that the presence of an active additive with antibacterial and antioxidant properties inhibits the breakdown of the fruit’s outer layer, which is an important barrier for the fruit pulp and protects it from mechanical, chemical, and biological factors. This allows the fruit to retain its freshness and nutritional value for a longer period.

Another factor that may indicate changes in fruit quality is firmness. Firmness is defined as the maximum force required to achieve a specified deformation during compression. However, considering that the tested blueberries varied in diameter, Young’s modulus was applied to assess the compression effect. Calculating this modulus requires data comprising the cross-sectional area measurements of the tested sample. It should also be noted that both parameters (firmness and Young’s modulus) reflect the tissue’s capacity to resist deformation. The Young’s modulus values for the tested blueberries are presented in Figure 7C. The results align with the trend of fruit mass loss, indicating that higher mass loss correlates with lower Young’s modulus values. All berries showed a reduction in Young’s modulus compared to fresh fruit. Similarly to mass loss, the lowest values were observed in unpackaged fruit, while blueberries packaged in film containing 3% cinnamic acid exhibited the highest Young’s modulus values. These Young’s modulus data clearly demonstrate a correlation between the concentration of the active additive in the films and the firmness of the blueberries. A similar effect of cinnamic acid on blueberry storage was observed in the work of Zasada et al. [21], where the chitosan-based materials containing ellagic acid and cinnamic acid were studied.

### 3.8. Antibacterial Properties

**Figure 8:**
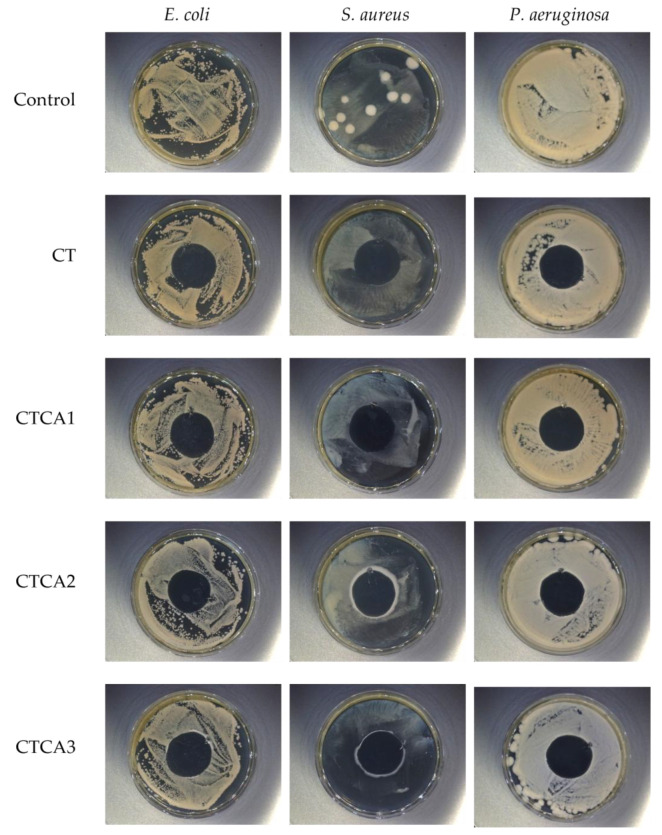
Antibacterial properties of the films (against *E. coli*, *S. aureus*, and *P. aeruginosa*) in accordance with ISO 20645.

In order to evaluate the antimicrobial properties of polymer materials containing cinnamic acid, analyses were carried out in accordance with ISO 20645 (Figure 8) and ISO 22196 standards (Table 2). Both methods allowed for the determination of antibacterial activity, but they are based on different measurement mechanisms, which made it possible to obtain complementary data on the effectiveness of cinnamic acid under various conditions of contact with microorganisms.

The results obtained using the ISO 22196 method, which involves the quantitative determination of the number of live bacterial cells on the surface of the material after 24 h of incubation, also confirmed the effectiveness of cinnamic acid. The reduction in the number of bacteria compared to the control sample (unmodified) was 2.4–2.6 log units for *S. aureus*, 1.8–2.3 log for *E. coli*, and 2.1–2.6 log for *P. aeruginosa* (Table 2). It should be noted that a very high level of activity was observed for all samples containing cinnamic acid.

When comparing the results of both methods, it can be seen that ISO 20645 better reflects the ability of the active substance to migrate into the environment (diffusion effect), while ISO 22196 provides information on the actual antimicrobial activity on the surface of the material (contact effect). In the case of cinnamic acid, effectiveness was observed in terms of both diffusion and sustained surface activity. This confirms the usefulness of CA as an active ingredient in polymer systems with a wide range of applications, from active packaging to medical device components.

The results of this study also suggest that the effectiveness of cinnamic acid is significantly dependent on its concentration and the method of its incorporation into the polymer matrix. In single-phase systems, faster CA release and more pronounced inhibition zones were observed, while in layered or multi-phase systems, surface action dominated, confirmed by higher log values of bacterial reduction in the ISO 22196 test. The results obtained are consistent with previous literature reports indicating the high antimicrobial activity of CA and its derivatives in polymer composites [18,20].

The use of natural bioactive compounds, such as cinnamic acid (CA), in polymer materials is a promising strategy for the development of active packaging materials and biopolymers with antimicrobial properties. Cinnamic acid, as a compound with documented antibacterial and antifungal properties, is gaining importance as a component that modifies the functional properties of plastics.

Cinnamic acid belongs to phenolic acids with proven activity against a wide spectrum of microorganisms, including Gram-positive (*Staphylococcus aureus*) and Gram-negative (*Escherichia coli*, *Pseudomonas aeruginosa)* bacteria, as well as yeasts and molds. According to the literature [47,48], the antimicrobial mechanism of cinnamic acid primarily involves disruption of the microbial cell membrane, which leads to leakage of vital intracellular components and loss of membrane potential, which is fatal to the cell. Moreover, cinnamic acid can also interfere with the synthesis or function of nucleic acids and proteins, further inhibiting microbial growth and viability [49]. Some studies also indicate that cinnamic acid and its derivatives can inhibit biofilm formation, which is crucial for bacterial resistance and persistence [50].

Numerous studies have shown that cinnamic acid (CA) can impart antimicrobial activity to packaging materials. However, this effectiveness is strongly dependent on the type of polymer matrix, the method of application of the compound, and its ability to be released when in contact with food. In the case of starch matrices, CA exhibits high antimicrobial activity, particularly against *Listeria innocua*, whose numbers were reduced by more than 7 log CFU/mL at a CA content of 3% [18]. The effectiveness of the action is related to the hydrophilic nature of starch, which facilitates the diffusion of phenolic compounds into the aqueous environment [18]. Good results were also achieved in food films, with 2% CA effectively inhibiting the growth of microflora in melon and poultry meat [51]. In PVA-based systems, cinnamic acid has demonstrated effective antibacterial activity, particularly against Gram-positive bacteria, while maintaining film transparency, which is desirable in fresh food packaging [22]. Similarly, biodegradable films made of alginate and pectin enriched with CA showed high efficacy against *S. aureus* and *E. coli*, as well as a significant reduction in microflora in beef, by over 84% after 5 days of storage [20]. The PLA matrix behaves completely differently, despite the introduction of up to 10% CA—it does not show significant biocidal activity. This is probably due to the strong binding of molecules in a rigid, non-swelling matrix and their low mobility, which limits the migration of the compound to the substrate [34].

Surface application techniques such as ethanol spraying or electrospinning proved to be a breakthrough. In the case of layered PLA/starch/PLA films with surface-applied CA, a strong antimicrobial effect was observed—a reduction of *L. innocua* by more than 7 log and *E. coli* by more than 2 log CFU/mL [19]. CA proved to be more effective than FA, and Gram-positive bacteria were more sensitive than Gram-negative bacteria, confirming previous observations [19,21].

In some cases, such as chitosan films with CA, moderate efficacy was observed against *S. aureus* and *P. aeruginosa*, but limited efficacy against *E. coli* [21]. The effect was based on the synergy of phenolic additives and chitosan, which itself has antimicrobial properties. Interestingly, some modifications of PET materials using CA derivatives did not show typical bactericidal activity, but modulated quorum sensing mechanisms, limiting, among other things, the production of virulence factors in *P. aeruginosa* [52]. This approach is in line with modern antimicrobial strategies aimed not at killing bacteria, but at disrupting their communication and virulence.

The biocidal effectiveness of active materials containing cinnamic acid depends not only on the type and concentration of the compound, but above all on the characteristics of the matrix and the method of its application. Hydrophilic matrices, such as starch or alginate, promote the release and action of phenolic active agents, while PLA requires a surface application strategy. These compounds show potential not only as classic antimicrobial agents but also as modulators of pathogen behavior, which opens up new avenues of research in the field of active packaging materials.

## 4. Conclusions

The developed polymer films based on cellulose acetate propionate (CAP) with the addition of Tween 80 and cinnamic acid demonstrated a range of beneficial properties relevant to modern food packaging. The incorporation of cinnamic acid significantly improved the material’s UV barrier properties as well as antibacterial activity, which directly contributed to the prolonged freshness of stored blueberries. At the same time, the presence of cinnamic acid did not significantly affect the transparency of the films, allowing the contents of the packaging to remain visible to consumers. Therefore, the obtained composites represent a promising alternative to traditional plastic packaging, combining enhanced UV protection, antibacterial activity, and maintained visual aesthetics, which can help reduce food waste.

## Figures and Tables

**Table 1:** Comparative summary of ISO 20645 and ISO 22196 methods.

Parameter	ISO 20645	ISO 22196
Type of assessment	Qualitative	Quantitative
Tested organisms	*Staphylococcus aureus* (ATCC 6538), *Escherichia coli* (ATCC 11229)	*Staphylococcus aureus* (ATCC 6538P) *Escherichia coli* (ATCC 8739)
Sample type	Non-leaching materials	Non-porous plastic or coated surfaces
Method principle	Agar diffusion (zone of inhibition and contact activity)	Direct inoculation and recovery of bacteria after contact
Evaluation criteria	Presence/absence and size of inhibition zone; Growth under sample	Logarithmic reduction in CFU after 24 h
Incubation conditions	37 ± 1 °C, 18–24 h	35 ± 1 °C, ≥ 90% RH, 24 h-120 h
Result expression	Zone diameter and qualitative classification	Log reduction value (e.g., ≥ 2 log = significant activity)
Interpretation of results	Zone around = leaching activity; Only under = contact activity	Numerical efficacy against baseline/control surface

**Table 2:** Antimicrobial activity of samples in relation to the control sample against pathogenic strains.

Bacteria Strains	Samples	R	% Reduction	Antibacterial Efficacy
*E. coli* (ATCC 8739P)	CT	-	-	-
	CTCA1	1.8	>90.0	satisfactory
	CTCA2	2.0	>99.0	very good
	CTCA3	2.3	>99.0	very good
*S. aureus* (ATCC 65388)	CT	-	-	-
	CTCA1	2.4	>99.9	very good
	CTCA2	2.5	>99.9	very good
	CTCA3	2.7	>99.9	very good
*P. aeruginosa* (ATCC 8739)	CT	-	-	-
	CTCA1	2.1	>99.0	very good
	CTCA2	2.3	>99.0	very good
	CTCA3	2.6	>99.0	very good

## Data Availability

The original contributions presented in this study are included in the article; further inquiries can be directed to the corresponding author.
